# Supplementary material for: HIVprotI: an integrated web based platform for prediction and design of HIV proteins inhibitors
Source: J Cheminform. 2018 Mar 9;10:12. doi: 10.1186/s13321-018-0266-y (PMC5845081; doi:10.1186/s13321-018-0266-y)
Supplement: Supplementary file 2 — Additional file 2. Source code of HIVProtI web server. [file 13321_2018_266_MOESM2_ESM.zip › HIVprotI_Source-code/hivprotI/weka-3-6-6/doc/weka/core/converters/package-frame.html]

weka.core.converters


weka.core.converters

Interfaces 
  
*BatchConverter*
  
*DatabaseConverter*
  
*FileSourcedConverter*
  
*IncrementalConverter*
  
*Loader*
  
*Saver*
  
*URLSourcedLoader*

Classes 
  
AbstractFileLoader
  
AbstractFileSaver
  
AbstractLoader
  
AbstractSaver
  
ArffLoader
  
ArffLoader.ArffReader
  
ArffSaver
  
C45Loader
  
C45Saver
  
ConverterUtils
  
ConverterUtils.DataSink
  
ConverterUtils.DataSource
  
CSVLoader
  
CSVSaver
  
DatabaseConnection
  
DatabaseLoader
  
DatabaseSaver
  
LibSVMLoader
  
LibSVMSaver
  
SerializedInstancesLoader
  
SerializedInstancesSaver
  
SVMLightLoader
  
SVMLightSaver
  
TextDirectoryLoader
  
XRFFLoader
  
XRFFSaver
